# Debonding force and shear bond strength of an array of CAD/CAM-based customized orthodontic brackets, placed by indirect bonding- An *In Vitro* study

**DOI:** 10.1371/journal.pone.0202952

**Published:** 2018-09-11

**Authors:** Ha-Na Sha, Sung-Hwan Choi, Hyung-Seog Yu, Chung-Ju Hwang, Jung-Yul Cha, Kwang-Mahn Kim

**Affiliations:** 1 Department of Orthodontics, Institute of Craniofacial Deformity, College of Dentistry, Yonsei University, Seoul, Korea; 2 Department and Research institute of Dental Biomaterials and Bioengineering, College of Dentistry, Yonsei University, and Brain Korea 21 PLUS project, Seoul, Korea; National Taiwan University, school of dentistry, TAIWAN

## Abstract

Based on three-dimensional scanning and computer-aided design and computer-aided manufacturing (CAD/CAM) techniques, customized bracket systems are increasingly used. However, data remain limited regarding customized bracket design, characteristics, and stability. This study was undertaken to evaluate the design, bond strength, and residual adhesives of four different CAD/CAM customized brackets that were attached to human tooth specimens by indirect bonding. Thirty extracted human upper premolars were divided into five groups: Group 1, preadjusted self-ligating labial metal bracket; Group 2, lingual self-ligating metal injection molding customized bracket; Group 3, gold-casted lingual customized bracket; Group 4, labial self-ligating milled customized bracket; Group 5, labial customized resin base bracket. Except in Group 1, premolar specimens were scanned via model scanner, and the images were sent to each manufacturing company to fabricate customized brackets and transfer trays/jigs. Debonding force (DF; N) was measured by Instron universal testing machine and shear bond strength (SBS; MPa) was calculated via dividing DF by bonding area. Adhesive remnants were analyzed via stereo microscopic images. Group 2 (196.90±82.75 N) exhibited significantly higher DF than Group 1 (62.77±12.65 N); other groups exhibited similar DFs, compared with Group 1. No customized bracket groups exhibited significant differences in SBS, relative to Group 1 (6.73±1.36 MPa). However, SBS in Group 5 (11.46±7.22 MPa) was significantly higher than in Group 3 (3.58±2.14 MPa). Group 3 had significantly lower ARI scores than other groups (P<0.05). Customized brackets exhibited large deviations in DF and SBS; all customized bracket systems exhibited DF that was equivalent or superior to that of preadjusted brackets, even when placed by indirect bonding.

## Introduction

Computer-aided design and computer-aided manufacturing (CAD/CAM) systems have had great impacts on the efficiency and accuracy of dental treatment in recent years [1], as they have been implemented in both the dental laboratory and the clinic [2]. CAD/CAM systems facilitate higher quality of dental products along with reduced production time; in some cases, CAD/CAM systems have enabled restoration in patients with a single visit [3].

In orthodontics, this advanced technology facilitates both diagnosis and treatment, especially in complex cases, such as patients with multiple missing teeth or those with dentofacial deformities [4]. By using a CAD/CAM-based system, clinicians can generate different three-dimensional (3D) digital models, depending on treatment plans, and then choose the best treatment option [5, 6]. Furthermore, 3D virtual models are useful for communication among specialist clinicians, as well as with patients, who must approve the final treatment approach.

Based on the use of 3D images and the CAD/CAM technique, a customized bracket system has been introduced to move orthodontic treatment into the next era [7–11]. In previous studies that have compared conventional and CAD/CAM-based customized brackets, the customized system has demonstrated acceptable treatment outcome [12], enhanced patient comfort [11–13], and significantly reduced both total treatment time and the number of scheduled appointments [11].

Nevertheless, even though the CAD/CAM system may greatly enhance treatment, concerns remain in terms of high costs and technique sensitivity. Moreover, even though the computerized technique provides high-quality 3D volume images and bracket system design, discrepancies remain between the virtual plan and the final outcome [13–16]. Recently, the importance of precision dental treatment, using CAD/CAM-based customized bracket and wire systems, has been emphasized [13], but further studies are required to discern the capability and treatment outcome of these systems.

Many companies produce CAD/CAM-based customized brackets; therefore, the characteristics of each customized bracket vary depending on materials, design, and base topography. For instance, Harmony is constructed from Cr-Co alloy by metal injection molding (MIM); Incognito is fabricated from gold alloy with extended bracket bases; Insignia is produced via milling bracket slots with different torques of the target tooth; Orapix utilizes a virtual design model to build a customized resin base on the preadjusted bracket base. Moreover, each company provides their own specialized indirect bonding system, with a unique transfer tray or jig. Because of these different design characteristics, customized bracket systems are varied and more intricate than the preadjusted bracket system.

Since each customized bracket is designed using a virtual model, the indirect bonding method may provide an optimized bracket position. Previous studies have indicated that the bond strength from indirect bonding may be lower than that of direct bonding; however, this difference is not statistically significant [17–19]. Thus far, there has been no study to investigate the bonding ability of customized bracket systems that are placed using an indirect bonding method.

Bracket failure during orthodontic treatment may cause unwanted biomechanical effects, prolong treatment time, and even hurt the patient. Because of the absence of substitute brackets, a customized bracket must be rebonded when it fails; consequently, the shear bond strength decreases [20]. Therefore, a robust bonding ability of customized bracket systems is necessary to ensure their long-term clinical performance.

Other crucial factors that affect bond strength are the bracket base design, bracket surface characteristics, and bracket bonding area [21–25]. There are various types of customized bracket bases, such as composite resin base, occlusal clasp extension, surface treatment with sandblasting, and an encircled bracket base design. The size of the customized bracket base also varies depending on the tooth morphology. Therefore, variations in bond strength exist among different bracket systems, as well as within a single customized bracket system.

Because of the divergent design and limited clinical knowledge of customized bracket systems, the aim of this study was to investigate the bonding strength of four customized bracket systems that were placed using an indirect bonding method. Further, it was designed to determine whether CAD/CAM-based customized brackets could provide equivalent bonding force to that provided by preadjusted brackets.

## Materials and methods

### Collection and scanning of tooth specimens

A total of 30 upper premolars were extracted from patients who were undergoing orthodontic treatment, according to their treatment plans. The use of extracted human teeth was performed in accordance with the guidelines of the ethics committee of the Yonsei University dental hospital and all the patients signed informed consent forms before enrollment. The protocol for this study was approved by the institutional review board (IRB) of the Yonsei University (2-2018-0024).Teeth were cleaned, and soft tissue was removed by periodontal curette immediately after extraction; then, teeth were stored in a 50°C refrigerator, within a physiologic saline solution, for ≤3 months before testing. The inclusion criteria were as follows: (1) teeth in which the crowns did not exhibit any large defect, restoration, or crack line, (2) teeth that had never been treated with any chemical agent, such as formalin or hydrogen peroxide, (3) teeth in which labial and lingual surfaces were never previously bonded to any bracket, and (4) teeth in which the crown size was normal, as confirmed by measurement of crown height and width.

The teeth were separated into five groups of six teeth each. Of the five groups, Group 1 (control group) underwent direct bonding with a preadjusted bracket (Clippy M, Tomy, Tokyo, Japan); Group 2 underwent indirect bonding with the Harmony bracket (American Orthodontic, Sheboygan, WI, USA); Group 3 underwent indirect bonding with the Incognito bracket (3M Unitek, Monrovia, Calif, USA); Group 4 underwent indirect bonding with the Insignia bracket (Ormco, Orange, CA, USA); and Group 5 underwent indirect bonding with the Orapix bracket (Orapix, Seoul, Korea). The preadjusted, Insignia, and Orapix brackets are labial brackets; the Harmony and Incognito brackets are lingual brackets.

### Design and fabrication of customized brackets and transfer trays/jigs

Teeth were scanned with an intraoral scanner (Trios 3, OrthosAnalyzer 3 Shape, Copenhagen, Denmark) to obtain 3D images that were then sent to each company to produce the customized brackets.

#### Group 2 (Harmony)

After virtual design, a large customized pad was created on the lingual surface of each tooth. The self-ligature slot was designed using a virtual model with torque and in-out dimensions and was positioned on each customized pad by a customized adapter. Then, the MIM method was used to produce each bracket.

#### Group 3 (Incognito)

On the virtual model, the bracket base and customized bracket were designed. The rapid prototyping wax printer base on the virtual image was used to produce the bracket wax pattern, which was then cast in a gold alloy. The bracket base was sandblasted and coated with silane.

#### Group 4 (Insignia)

After the virtual design location was confirmed, a customized slot was cut into the bracket at the desired position. The bracket base was a conventional mesh base that was modified with a customized slot to generate the prescribed bracket. The relationship between tooth and bracket was recorded to generate the 3D printed transfer jig that enabled transfer of the actual bracket to the patient mouth [5].

#### Group 5 (Orapix)

**Fig 1:**
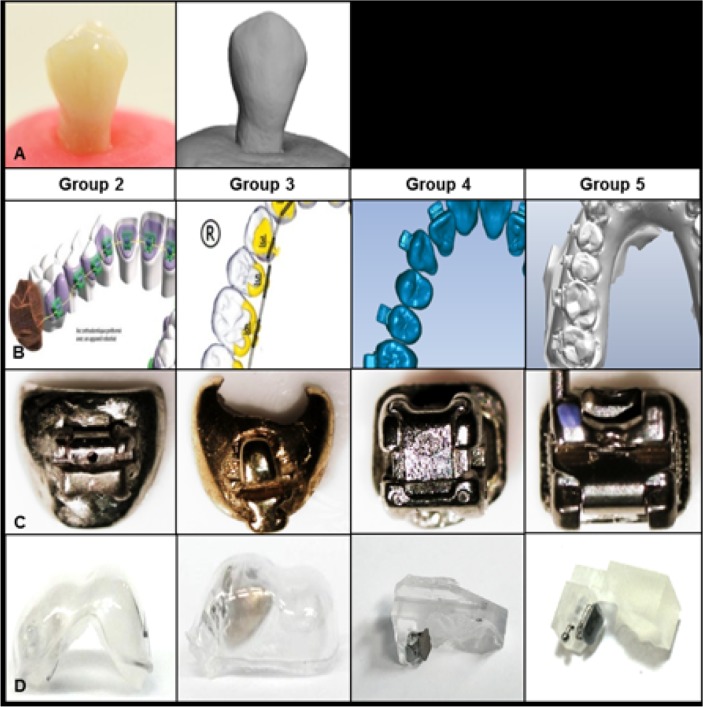
The design and fabrication of customized brackets. A. Scanning and reconstruction of virtual tooth imagine B, Virtual set up and bracket design; C, Bracket fabrication; D, Transfer tray (Groups 2 and 3)/ Transfer jig (Groups 4 and 5). Group 2, Harmony; Group 3, Incognito; Group 4, Insignia; Group 5, Orapix.

The virtual model was set up with ideal arch form, then constructed using the 3Txer program, (Orapix, Seoul, Korea); the virtual brackets and transfer jigs were placed on the ideal virtual model. The real transfer jigs were printed using a stereolithographic rapid-prototyping machine (Viper 2, 3D system, Circle Rock Hill, SC, USA). Using the transfer jig, adhesive paste (Transbond XT, 3M Dental Products, St. Paul, MN, USA) was applied to the preadjusted bracket (Clippy M, Tomy, Japan) to fill the space between bracket base and tooth surface. After curing to polymerize the resin, the customized base was completed (Fig 1A, 1B, 1C and 1D).

### Bracket stem length evaluation

Lateral stereo microscope (Olympus SZ61, Olympus Austria, Vienna, Austria) images of each bracket were obtained to measure the stem length, which is the distance between the wing and bracket base.

### SEM (Scanning Electron Microscopy)

One extra bracket, which was obtained from each customized company, was cleaned ultrasonically using acetone, then fixed in 10% formaldehyde and treated with gold-palladium, using HITACHI E-1010 ion sputter. Each bracket was then observed using HITACHI S– 3000N scanning microscope at 20×, 150×, and 1000× magnifications.

### Bracket base surface dimension calculation

**Fig 2:**
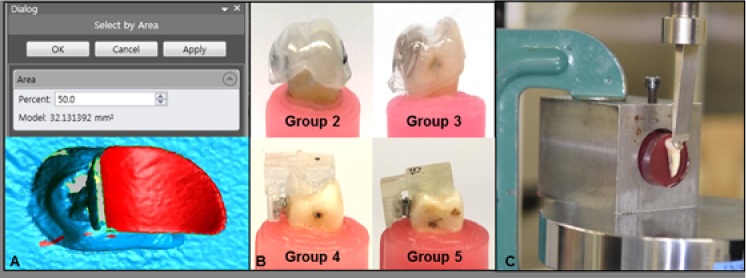
Bracket base calculation, indirect bonding and debonding force test. A. Measurement of bracket bonding area by 3D analysis program; B. Indirect bonding on tooth with transfer tray/jig; C. Measurement of debonding force by Instron machine.

The bracket base surface area was calculated from the 3D surface scanned data. Each bracket was scanned by a 3D model scanner (Identica-hybrid, Medit HQ, Seoul, Korea). The 3D images were saved as STL files in the Exocad program (BEGO Medical GmbH, Bremen, Germany) and the base surface dimension was measured in mm^2^ via 3D design software (Geomagic, 3D SYSTEMS, Rock Hill, SC, USA) (Fig 2A).

### Bonding

Depending on the bracket system, either the labial or lingual surface of each tooth underwent 37% phosphoric acid treatment for 15 seconds, then was rinsed completely. Before bracket bonding, teeth surfaces were dried with air spray until they reached a chalky-white appearance.

Dual cure self-adhesive resin cement (RelyX^TM^ U200, ESPE Dental Products, 3M Deutschland GmbH, Neuss, Germany) was used to coat the entire surface of the bracket base with a micro-brush; excess cement was removed until only a thin and even layer of cement remained. The bracket and transfer tray/jig were seated with firm pressure on the teeth, followed by light curing for 3 seconds on all four sides. After curing, the transfer tray/jig was removed, and excess cement was removed by bur (Fig 2B).

All specimens were stored in a 37°C water bath for 24 hours before the debonding test, according to the DIN ISO 3696 recommendation. Before the debonding test, teeth were embedded in a 30-mm diameter, 40-mm high cylindrical shape resin block (Polycoat, AEKYUNG Chemical Co., Ltd., Seoul, Korea). A positioning jig with square wire (0018X0018 SS, Ormco, CA, USA) was connected to the embedded box and ligated with the bracket specimen to ensure that the bracket slot was perpendicular to the direction of shear bond force, as well as to construct an identically inclined bracket slot during the shear bond test.

### Shear test and failure analysis

The debonding force of each bracket (in N) was measured by the Instron Universal Testing Machine (3366 Single Column Testing System, Instron, Norwood, MA, USA). The samples were aligned with the bonding surface parallel to the blade; the force point was set at the middle of the bracket base and wings. Due to the customized torque design of the Insignia, the stem depth was different for each Insignia bracket. Therefore, the force point was set to close to the slot, imitate the occlusal force encountered in the clinic. A debonding force was applied by moving the shear blade in an occlusogingival direction, with a crosshead speed of 1 mm/min. The shear bond strength (N/mm^2^) was calculated by dividing the debonding force by the surface area of the bonding base (Fig 2C).

After debonding of the bracket, the surfaces of teeth and bracket base were photographed and examined by stereo microscopy (Olympus SZ61, Olympus Austria, Vienna, Austria) to characterize the bonding failure interface. The residual adhesives on the tooth surface were assessed by the adhesive remnant index (ARI). In ARI scoring, 0 = no bonding resin remained on the tooth; 1 = less than 50% of bonding resin remained on the tooth; 2 = more than 50% of bonding resin remained on the tooth; 3 = all bonding resin remained on the tooth.

One bracket base of each group was taken by Scanning Electron Microscope to observe the pattern of debonding break leakage.

### Statistical analysis

One-way analysis of variance and Tukey’s test were used to determine the statistical significance of any intergroup differences in the mean debonding force, shear bond strength, and bonding area. The stem length of the brackets was analyzed by a Kruskal-Wallis H test, with a post-hoc test to determine the significant differences between groups. A Kruskal-Wallis ANOVA, with post-hoc pairwise comparisons, was used to evaluate the statistical significance of any coupled data differences among the control group, customized lingual brackets, and customized labial brackets. The Kruskal-Wallis H test was used to investigate the statistical significance of the ARI among the different surface conditioning methods, and the Mann-Whitney U test was performed for multiple comparisons. The significance level was set at p = 0.05. SPSS, version 23, (IBM, Armonk, NY, USA) was used for all statistical analyses.

## Results

### Stem length

The mean stem lengths of each group were 0.62 mm (SD 0.04), 0.7 mm (SD 0.17), 0.63 mm (SD 0.05), 1.12 mm (SD 0.32), and 1.03 mm (SD 0.05) for preadjusted, Harmony, Incognito, Insignia, and Orapix brackets, respectively. The stem lengths of Insignia and Orapix brackets were significantly longer from the stem lengths of preadjusted and Incognito brackets (p <0.05).

### SEM (Scanning Electron Microscopy) before bonding

**Fig 3:**
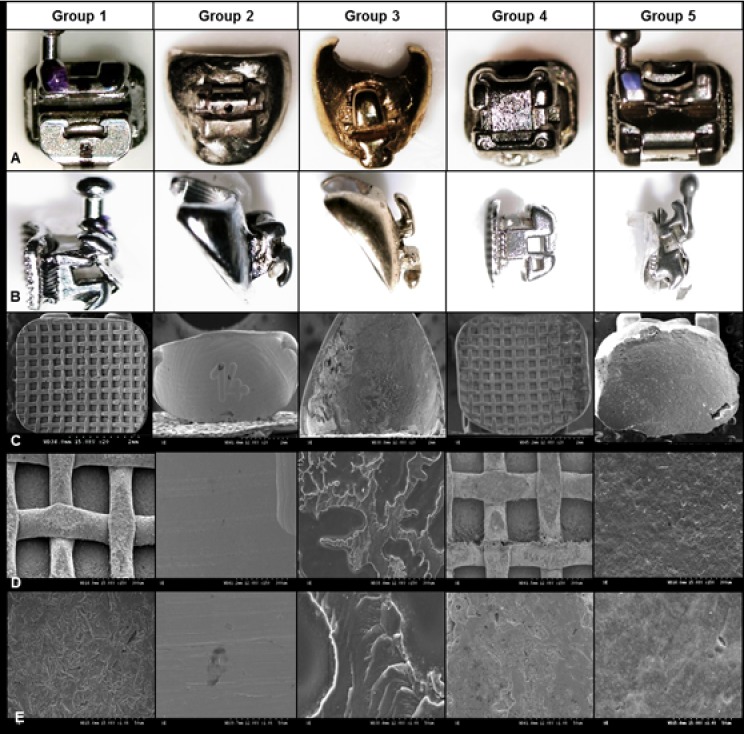
Stereo microscope and SEM imagines of bracket specimens. Stereo microscopic frontal and lateral imagines of bracket specimens and scanning electron microscopy imagines of bracket bases. A. Frontal view; B. Lateral view; C. Bracket bases (20×); D. Bracket bases (150×); E. Bracket bases (1000×). Group 1, preadjusted bracket; Group 2, Harmony; Group 3, Incognito; Group 4, Insignia; Group 5, Orapix.

The preadjusted and Insignia brackets exhibited a mesh base; the Harmony bracket revealed a smooth surface; the Incognito bracket had an irregular base surface caused by sandblasting and chemical coating; the base of the Orapix bracket was resin. In addition to the structure of the base, the SEM photos revealed differences in the appearance of each customized bracket system (Fig 3).

### Bracket base area

The mean base areas of the groups were 9.33 mm^2^ (SD 0.01), 32.35 mm^2^ (SD 1.37), 34.91 mm^2^ (SD 0.48), 10.20 mm^2^ (SD 0.26), and 11.82 mm^2^ (SD 0.48) for preadjusted, Harmony, Incognito, Insignia, and Orapix brackets, respectively. The preadjusted and Insignia brackets exhibited similar bracket size and were both significantly smaller than other brackets (p < 0.05). The Harmony, Incognito, and Orapix brackets also revealed significant differences in base size, compared with each other (p < 0.05).

### Debonding forces and shear bond strength

**Fig 4:**
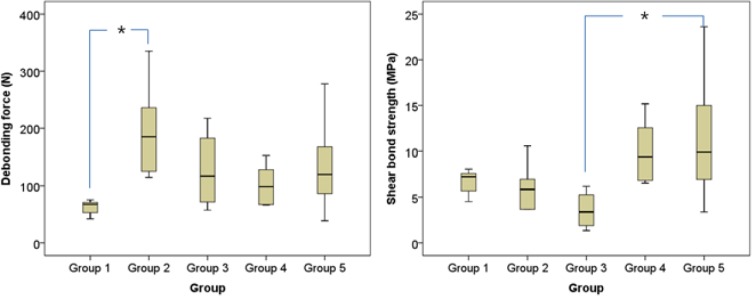
Box slot of debonding force and shear bonding strength. The bottom and top of the boxes are the 25^th^ and 75^th^ percentile respectively of debonding forces and SBS for each group. The band near the middle of the box denotes the median. Upper and lower horizontal lines outside the box represent maximum and minimum values within a 1.5 interquartile range. The superscripts indicate differences in debonding forces and shear bond strength, according to Tukey’s HSD test, at P = 0.05. Group 1, preadjusted bracket; Group 2, Harmony; Group 3, Incognito; Group 4, Insignia; Group 5, Orapix.

Descriptive statistics regarding the debonding force and shear bond strength for all groups are shown in Table 1, Table 2, and Fig 4. Statistically significant differences in debonding force were found between preadjusted and Harmony brackets, which were 62.77 N (SD 12.65) and 196.90 N (SD 82.75), respectively. Further, statistically significant differences in shear bond strength were found between Incognito and Orapix brackets, which were 3.58 N (SD 2.14) and 11.46 N (SD 7.22), respectively (p < 0.05).

**Table 1:** Mean, stand deviation (SD), and range of debonding force values (N).

Group	Minimum	Maximum	Mean	SD
1*	42.2	75.4	62.77	12.65
2*	114.7	335	196.9	82.75
3	57.4	217.5	127.2	71.04
4	66.49	152.64	101.98	34.02
5	38.7	277.7	134.98	83.82

*Group 2 showed significant greater debonding force than Group 1(P < .05) by Tukey test.

Group 1, preadjusted bracket; Group 2, Harmony; Group3, Incognito; Group 4, Insignia; Group 5, Orapix.

**Table 2:** Mean, standard deviation (SD), and range of shear bond strength values (MPa).

Group	Minimum	Maximum	Mean	SD
1	4.52	8.08	6.73	1.36
2	3.66	10.57	6.1	2.61
3*	1.34	6.2	3.58	2.14
4	6.56	15.17	9.99	3.36
5*	3.38	23.63	11.46	7.22

*Group 5 showed significantly greater SBS than Group 3 (P < .05) by Tukey test.

Group 1, preadjusted bracket; Group 2, Harmony; Group3, Incognito; Group 4, Insignia; Group 5, Orapix.

### Comparisons among control, customized lingual, and customized labial brackets

The bonding area of the lingual brackets was significantly larger than the bonding areas of either the labial or the preadjusted brackets. Preadjusted brackets exhibited significantly reduced debonding force (62.77±12.65), compared with customized lingual brackets (169.02±82.35). Labial brackets (10.72±5.42) exhibited significantly increased shear bond strength, compared with lingual brackets (5.09±2.65) (p < 0.05) (Table 3).

**Table 3:** Comparison of area, debonding force and shear bond strength between the control, lingual and labial brackets.

Category	Group	Number	Mean	SD
**Area(mm**^**2**^**)**	Control*	6	9.33	0.01
** **	Lingual^μ^	10	33.37	1.69
** **	Labial*	12	11.01	0.92
**Force(N)**	Control*	6	62.77	12.65
** **	Lingual^μ^	10	169.02	82.35
** **	Labial^μ^	12	118.48	63.38
**SBS(MPa)**	Control* ^μ^	6	6.73	1.36
	Lingual*	10	5.09	2.65
	Labial^μ^	12	10.72	5.42

Control, preadjusted bracket; Lingual, Incognito and Harmony; Labial, Insignia and Orapix.

The different superscripts indicated the statistic difference at p< 0.05

### Adhesive remnant index

The amount of residual adhesive on the tooth surface was evaluated by ARI scores, as shown in Table 4. Only Incognito brackets exhibited significantly lower scores (2.5), compared with other brackets (p < 0.05).

**Table 4:** Adhesive remnant index (ARI) scores on the teeth surface after debonding.

			ARI		
Group	0	1	2	3	N
1^A^	0(0)	1(17)	5(83)	0(0)	6
2^A^	0(0)	2(33)	4(67)	0(0)	6
3^B^	4(100)	0(0)	0(0)	0(0)	4
4^A^	0(0)	2(33)	3(50)	1(17)	6
5^A^	1(17)	1(17)	2(33)	2(33)	6

Values are presented as number (%)

0 = no bonding resin remaining on the tooth; 1 = less than 50% of bonding resin remaining on the tooth; 2 = more than 50% of bonding resin remaining on the tooth; 3 = all bonding resin remaining on the tooth.

Differing superscript letters indicate differences between groups according to Kruskal-Wallis and Mann-Whitney tests (p < .05).

Group 1, preadjusted bracket; Group 2, Harmony; Group3, Incognito; Group 4, Insignia; Group 5, Orapix.

**Fig 5:**
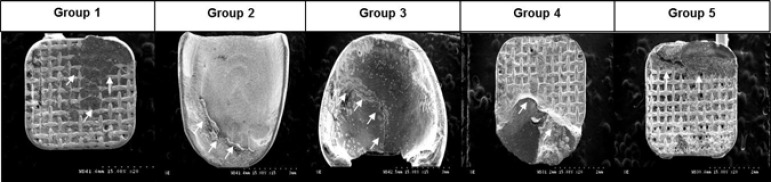
SEM imagines of bracket base after debonding. The resin cement remaining on the bracket base was pointed by the white arrows. The resin cement penetration within metal bracket mesh are shown in group 1, 4, and 5. Group 5 showed the most air voids compared with group 1 and 4. Group 2 showed a small amount on the bottom of bracket. Although a break line was evident, the resin cement completely covered all the surface of the bracket base in group 3. Group 1, preadjusted bracket; Group 2, Harmony; Group 3, Incognito; Group 4, Insignia; Group 5, Orapix.

The SEM photos of debonding bracket base were showed in Fig 5. Coincident with the ARI result, Incognito showed most of resin attached on the bracket surface. The resin cement remaining on mesh type bracket base, such as, preadjusted bracket, Insignia, and Orapix were engaged into the mesh and Orapix showed the most air voids. Harmony showed a smooth surface similar to the prebonded bracket base surface with only a small part of resin cement remnant on the bottom area.

## Discussion

There are many different experimental factors that might influence bonding ability. To reduce the effects of these factors on our results, the DIN 13990 standard for bonding force examination was utilized to standardize the experimental protocol in this study. Since upper premolars are more consistent in shape and size than lower premolars, all samples comprised upper premolars, which were separated by crown height and width to equalize the sample size [26].

A customized bracket is positioned within the virtual or manual design model based on treatment planning by clinician. The indirect bonding method, conducted with transfer tray/jig systems, provide an easier and more precise bonding process, especially for lingual brackets.

Recently, with the innovation of novel bonding agents, the bond strengths of the indirect and direct bonding methods have become similar for preadjusted bracket systems [17–19]. The value of the debonding force in all customized bracket systems that were included in this study was higher than the debonding force in the preadjusted bracket, which utilized a direct bonding method. Thus, the indirect bonding method did not have a negative effect on debonding force in the customized bracket system.

Divergent designs existed between brackets in this experiment, which could significantly impact the bonding force. Customized bracket systems are converging via CAD/CAM techniques with 3D printing. Depending on the design concept in each customized bracket system, the companies use different materials and manufacturing methods such as casting, milling, and resin building to fabricate their product; therefore, customized brackets exhibit disparate forms from company to company.

Scanning electron microscopic analysis also revealed different characteristics in each bracket base surface. The mesh base in the preadjusted bracket is one of the methods used to increase mechanical retention [27]. In customized bracket systems, only the Insignia bracket used a mesh base; other companies applied different designs to provide adequate bond strength, such as increasing bonding surface, occlusal clasps, and sandblasting to the bracket base [28, 29]. The exception to mechanical retention was the silane coating, which improved chemical retention in the Incognito system [27, 29–32]. With these special base treatments and large bonding surfaces, the customized bracket systems demonstrated high debonding forces; however, the shear bond strength represented an opposite pattern, especially within the lingual customized bracket systems, such as Incognito and Harmony, which exhibit >3-fold larger bonding area than the preadjusted and labial customized brackets. Both lingual customized bracket groups exhibited greater debonding force (in N), but lower shear bond strength (in MPa), compared with the preadjusted and labial customized bracket groups. Thus, we concluded that although these brackets exhibited high debonding force, their shear bond strength diminished because of the greater bonding area. However, the extended bonding surface might improve the bracket fitness and simplify the bonding process, which remains necessary and important in lingual bracket systems.

In terms of ARI, except for the Incognito bracket, all customized bracket groups showed no significant differences in intergroup comparisons, which indicated that most failures happened between the bracket base and adhesive. Incognito brackets revealed an ARI result that was significantly lower than that of other brackets, which indicated that most failures happened between the enamel and adhesive. Our results were consistent with previous studies, which demonstrated that special conditioning tests, such as sandblasting and silane coating, could enhance the bonding ability between the bracket base and adhesive [28, 32, 33].

Base on each tooth’s morphology, as well as the torque or stem length of the customized bracket for each treatment plan, deviations were found within the customized brackets that were produced for each group. To unify the experiment, the bracket slot was aligned perpendicular to the blade during the debonding test. However, because of the individual torque of each bracket, different configurations occurred between the bracket base and tooth surface. These assorted configurations, combined with the varied bonding area and the individual bracket prescription, led to increased deviations in debonding force.

The Insignia had a longer stem length with larger standard deviation (1.12 ± 0.32 mm) because of the manner in which the individual treatment prescription is designed on the stem and the slot of the Insignia bracket. To emulate the chewing force in the patient’s mouth, we arranged the blade close to the slot, rather than the bracket base. In this condition, the shear bonding moment can be affected by the stem length of the individual bracket, which may have resulted in the large standard deviation in bond strength [34].

Orapix constructs the customized resin base on a preadjusted bracket. Despite use of the same bracket between Orapix and preadjusted bracket groups, bond strength exhibited a greater deviation in Orapix brackets, compared with preadjusted brackets. The customized resin filled the space between the surface of the target teeth on the set up model and the preadjusted bracket base. Then, the shape and thickness of the customized resin base was modified according to the different tooth morphology and treatment prescription. Therefore, discrepancy in resin base thickness within the same experimental group may arise from increased deviation of bond strength in Orapix brackets [35].

The limitations of this study include the limited selection of adhesive types and the small sample size. To control for incomplete polymerization resulting from a large bracket base, the dual cure adhesive was selected as a bonding agent for all groups in this study. Notably, other adhesive types, such as light curing and chemical curing, may reveal different results depending on bracket characteristics. The high cost of customized brackets is one of the primary reasons to restrict sample size; further, some companies required whole dentition to serve as a virtual model, thus fabricating whole brackets for each tooth in their system. Another limitation is the variability in the quality of human enamel, which may contribute to the large standard deviations found in bond strength test [36, 37]. Furthermore, two specimens were excluded because of wing deformation during the debonding test in the Incognito bracket group; this may have occurred because the gold alloy is too soft to withstand excessive force. Despite these limitations, this study provides the first comparative data regarding the bonding stability of customized bracket systems.

## Conclusions

The individual design and base morphology of each customized bracket system induced large deviations in DF and SBS; nevertheless, all CAD/CAM-based customized bracket systems that were assessed exhibited a debonding force that was higher than, or similar to, that of the conventional bracket system, even when placed by indirect bonding methods. However, *in vivo* conditions are much more complex than *in vitro* experiments; thus, our results should be applied carefully in clinical settings. Further clinical trials with larger sample sizes are warranted to clarify the findings of our study.

## Supporting information

S1 File Study data. Bonding area (mm^2^), stem length (mm), debonding force (N), and shear bond strength (MPa) for all specimens’ tests in the study.
(PDF)

## References

[1] Miyazaki T, Hotta Y, Kunii J, Kuriyama S, Tamaki Y. A review of dental CAD/CAM: current status and future perspectives from 20 years of experience. Dent Mater J. 2009;28(1):44–56. . 19280967 10.4012/dmj.28.44

[2] Chen J, Ahmad R, Suenaga H, Li W, Sasaki K, Swain M, et al Shape Optimization for Additive Manufacturing of Removable Partial Dentures—A New Paradigm for Prosthetic CAD/CAM. PLoS One. 2015;10(7):e0132552 10.1371/journal.pone.0132552 ; PubMed Central PMCID: PMCPMC4498620. 26161878 PMC4498620

[3] Davidowitz G, Kotick PG. The use of CAD/CAM in dentistry. Dental clinics of North America. 2011;55(3):559–70, ix. 10.1016/j.cden.2011.02.011 . 21726690

[4] Lonic D, Pai BC, Yamaguchi K, Chortrakarnkij P, Lin HH, Lo LJ. Computer-Assisted Orthognathic Surgery for Patients with Cleft Lip/Palate: From Traditional Planning to Three-Dimensional Surgical Simulation. PLoS One. 2016;11(3):e0152014 10.1371/journal.pone.0152014 ; PubMed Central PMCID: PMCPMC4803320. 27002726 PMC4803320

[5] Grauer D, Wiechmann D, Heymann GC, Swift EJ Jr. Computer-aided design/computer-aided manufacturing technology in customized orthodontic appliances. J Esthet Restor Dent. 2012;24(1):3–9. 10.1111/j.1708-8240.2011.00500.x . 22296689

[6] Camardella LT, Rothier EK, Vilella OV, Ongkosuwito EM, Breuning KH. Virtual setup: application in orthodontic practice. J Orofac Orthop. 2016;77(6):409–19. 10.1007/s00056-016-0048-y . 27595882

[7] Alford TJ, Roberts WE, Hartsfield JK Jr., Eckert GJ, Snyder RJ. Clinical outcomes for patients finished with the SureSmile method compared with conventional fixed orthodontic therapy. Angle Orthod. 2011;81(3):383–8. 10.2319/071810-413.1 ; PubMed Central PMCID: PMCPMC5161459. 21261488 PMC5161459

[8] Wiechmann D, Rummel V, Thalheim A, Simon JS, Wiechmann L. Customized brackets and archwires for lingual orthodontic treatment. Am J Orthod Dentofacial Orthop. 2003;124(5):593–9. 10.1016/S0889540603007169 . 14614428

[9] Schubert K, Halbich T, Jost-Brinkmann PG, Muller-Hartwich R. Precision of indirect bonding of lingual brackets using the Quick Modul System (QMS)(R). J Orofac Orthop. 2013;74(1):6–17. 10.1007/s00056-012-0122-z . 23299653

[10] Brown MW, Koroluk L, Ko CC, Zhang K, Chen M, Nguyen T. Effectiveness and efficiency of a CAD/CAM orthodontic bracket system. Am J Orthod Dentofacial Orthop. 2015;148(6):1067–74. 10.1016/j.ajodo.2015.07.029 . 26672713

[11] Weber DJ 2nd, Koroluk LD, Phillips C, Nguyen T, Proffit WR. Clinical effectiveness and efficiency of customized vs. conventional preadjusted bracket systems. Journal of clinical orthodontics: JCO. 2013;47(4):261–6; quiz 8. . 23660822

[12] Pauls AH. Therapeutic accuracy of individualized brackets in lingual orthodontics. J Orofac Orthop. 2010;71(5):348–61. 10.1007/s00056-010-1027-3 . 20963544

[13] Muller-Hartwich R, Jost-Brinkmann PG, Schubert K. Precision of implementing virtual setups for orthodontic treatment using CAD/CAM-fabricated custom archwires. J Orofac Orthop. 2016;77(1):1–8. 10.1007/s00056-015-0001-5 . 26753550

[14] Im J, Cha JY, Lee KJ, Yu HS, Hwang CJ. Comparison of virtual and manual tooth setups with digital and plaster models in extraction cases. Am J Orthod Dentofacial Orthop. 2014;145(4):434–42. 10.1016/j.ajodo.2013.12.014 . 24703281

[15] Grauer D, Proffit WR. Accuracy in tooth positioning with a fully customized lingual orthodontic appliance. Am J Orthod Dentofacial Orthop. 2011;140(3):433–43. 10.1016/j.ajodo.2011.01.020 . 21889089

[16] Gan N, Xiong Y, Jiao T. Accuracy of Intraoral Digital Impressions for Whole Upper Jaws, Including Full Dentitions and Palatal Soft Tissues. PLoS One. 2016;11(7):e0158800 10.1371/journal.pone.0158800 ; PubMed Central PMCID: PMCPMC4934918. 27383409 PMC4934918

[17] Menini A, Cozzani M, Sfondrini MF, Scribante A, Cozzani P, Gandini P. A 15-month evaluation of bond failures of orthodontic brackets bonded with direct versus indirect bonding technique: a clinical trial. Prog Orthod. 2014;15:70 10.1186/s40510-014-0070-9 ; PubMed Central PMCID: PMCPMC4279038. 25547461 PMC4279038

[18] Milne JW, Andreasen GF, Jakobsen JR. Bond strength comparison: a simplified indirect technique versus direct placement of brackets. Am J Orthod Dentofacial Orthop. 1989;96(1):8–15. . 2526578 10.1016/0889-5406(89)90223-0

[19] Swetha M, Pai VS, Sanjay N, Nandini S. Indirect versus direct bonding—a shear bond strength comparison: an in vitro study. J Contemp Dent Pract. 2011;12(4):232–8. . 22186856 10.5005/jp-journals-10024-1040

[20] Eslamian L, Borzabadi-Farahani A, Tavakol P, Tavakol A, Amini N, Lynch E. Effect of multiple debonding sequences on shear bond strength of new stainless steel brackets. J Orthod Sci. 2015;4(2):37–41. 10.4103/2278-0203.156027 ; PubMed Central PMCID: PMCPMC4427969. 26020036 PMC4427969

[21] MacColl GA, Rossouw PE, Titley KC, Yamin C. The relationship between bond strength and orthodontic bracket base surface area with conventional and microetched foil-mesh bases. Am J Orthod Dentofacial Orthop. 1998;113(3):276–81. . 9517718 10.1016/s0889-5406(98)70297-5

[22] Merone G, Valletta R, De Santis R, Ambrosio L, Martina R. A novel bracket base design: biomechanical stability. Eur J Orthod. 2010;32(2):219–23. 10.1093/ejo/cjp077 . 19892719

[23] Sharma-Sayal SK, Rossouw PE, Kulkarni GV, Titley KC. The influence of orthodontic bracket base design on shear bond strength. Am J Orthod Dentofacial Orthop. 2003;124(1):74–82. 10.1016/S0889540603003111 . 12867901

[24] Sung JW, Kwon TY, Kyung HM. Debonding forces of three different customized bases of a lingual bracket system. Korean J Orthod. 2013;43(5):235–41. 10.4041/kjod.2013.43.5.235 ; PubMed Central PMCID: PMCPMC3822063. 24228238 PMC3822063

[25] Wang WN, Li CH, Chou TH, Wang DD, Lin LH, Lin CT. Bond strength of various bracket base designs. Am J Orthod Dentofacial Orthop. 2004;125(1):65–70. 10.1016/S0889540603007364 . 14718881

[26] Hobson RS, McCabe JF, Hogg SD. Bond strength to surface enamel for different tooth types. Dent Mater. 2001;17(2):184–9. . 11163390 10.1016/s0109-5641(00)00068-3

[27] Kang DY, Choi SH, Cha JY, Hwang CJ. Quantitative analysis of mechanically retentive ceramic bracket base surfaces with a three-dimensional imaging system. Angle Orthod. 2013;83(4):705–11. 10.2319/100412-782.1 . 23270384 PMC8754031

[28] Kachoei M, Mohammadi A, Esmaili Moghaddam M, Rikhtegaran S, Pourghaznein M, Shirazi S. Comparison of multiple rebond shear strengths of debonded brackets after preparation with sandblasting and CO2 laser. J Dent Res Dent Clin Dent Prospects. 2016;10(3):148–54. doi: 10.15171/joddd.2016.024 ; PubMed Central PMCID: PMCPMC5025215. 27651880 10.15171/joddd.2016.024 PMC5025215

[29] Mair L, Padipatvuthikul P. Variables related to materials and preparing for bond strength testing irrespective of the test protocol. Dent Mater. 2010;26(2):e17–23. 10.1016/j.dental.2009.11.154 . 20074788

[30] Matinlinna JP, Lung CYK, Tsoi JKH. Silane adhesion mechanism in dental applications and surface treatments: A review. Dent Mater. 2018;34(1):13–28. 10.1016/j.dental.2017.09.002 . 28969848

[31] Atsu S, Catalbas B, Gelgor IE. Effects of silica coating and silane surface conditioning on the bond strength of rebonded metal and ceramic brackets. J Appl Oral Sci. 2011;19(3):233–9. 10.1590/S1678-77572011000300010 ; PubMed Central PMCID: PMCPMC4234335. 21625739 PMC4234335

[32] Jung MH, Shon WJ, Park YS, Chung SH. Effects of silanation time on shear bond strength between a gold alloy surface and metal bracket. Korean J Orthod. 2013;43(3):127–33. 10.4041/kjod.2013.43.3.127 ; PubMed Central PMCID: PMCPMC3694204. 23814707 PMC3694204

[33] Viwattanatipa N, Jermwiwatkul W, Chintavalakorn R, Nanthavanich N. The effect of different surface preparation techniques on the survival probabilities of orthodontic brackets bonded to nanofill composite resin. J Orthod. 2010;37(3):162–73. 10.1179/14653121043065 . 20805345

[34] Klocke A, Kahl-Nieke B. Influence of force location in orthodontic shear bond strength testing. Dent Mater. 2005;21(5):391–6. 10.1016/j.dental.2004.07.004 . 15826695

[35] Jain M, Shetty S, Mogra S, Shetty VS, Dhakar N. Determination of optimum adhesive thickness using varying degrees of force application with light-cured adhesive and its effect on the shear bond strength of orthodontic brackets: an in vitro study. Orthodontics (Chic). 2013;14(1):e40–9. doi: 10.11607/ortho.919 . 23646337 10.11607/ortho.919

[36] Brosh T, Strouthou S, Sarne O. Effects of buccal versus lingual surfaces, enamel conditioning procedures and storage duration on brackets debonding characteristics. J Dent. 2005;33(2):99–105. 10.1016/j.jdent.2004.08.005 . 15683890

[37] Willems G, Carels CE, Verbeke G. In vitro peel/shear bond strength evaluation of orthodontic bracket base design. J Dent. 1997;25(3–4):271–8. . 9175357 10.1016/s0300-5712(96)00007-3

