# Supplementary material for: Debonding force and shear bond strength of an array of CAD/CAM-based customized orthodontic brackets, placed by indirect bonding- An In Vitro study
Source: PLoS One. 2018 Sep 11;13(9):e0202952. doi: 10.1371/journal.pone.0202952 (PMC6133347; doi:10.1371/journal.pone.0202952)
Supplement: S1 File — Bonding area (mm2), stem length (mm), debonding force (N), and shear bond strength (MPa) for all specimens’ tests in the study. (PDF) [file pone.0202952.s001.pdf]

Category	Number	Group	depth	area	Newton	Mpa
Control	1	1	0.6	9.33	69.8	7.48
	2	1	0.6	9.32	70.9	7.6
	3	1	0.7	9.33	42.2	4.52
	4	1	0.6	9.34	53	5.68
	5	1	0.6	9.33	75.4	8.08
	6	1	0.6	9.33	65.3	7
Mean			0.6166667	9.33	62.766667	6.7266667
SD			0.0408248	0.0063246	12.649216	1.3565643
Harmony	1	2	0.8	31.69	335	10.57
	2	2	0.8	31.12	157.9	5.07
	3	2	0.8	31.24	114.7	3.67
	4	2	0.6	33.85	236.1	6.97
	5	2	0.4	34.28	125.4	3.66
	6	2	0.8	31.91	212.3	6.65
Mean			0.7	32.348333	196.9	6.0983333
SD			0.167332	1.3674124	82.747205	2.6061344
Incognito	1	3	0.6	35.06	57.4	1.34
	2	3	0.6	35.06	217.5	6.2
	3	3	0.6	35.31	86.1	2.44
	4	3	0.7	34.22	147.8	4.32
Mean			0.625	34.9125	127.2	3.575
SD			0.05	0.4764714	71.039191	2.1392444
Insignia	1	4	1.4	10.62	93.9	8.84
	2	4	1.6	10.05	152.64	15.17
	3	4	0.8	10.22	128.29	12.55
	4	4	0.9	9.84	67.33	6.84
	5	4	1.1	10.13	66.49	6.56
	6	4	0.9	10.32	103.22	10
Mean			1.1166667	10.196667	101.97833	9.9933333
SD			0.3188521	0.2638687	34.016579	3.35845
Orapix	1	5	1	11.82	91.6	7.75
	2	5	1.1	11.44	38.7	3.38
	3	5	1.1	11.17	167.4	14.99
	4	5	1	12.4	86.2	6.95
	5	5	1	11.75	277.7	23.63
	6	5	1	12.33	148.3	12.03
Mean			1.0333333	11.818333	134.98333	11.455
SD			0.0516398	0.4833805	83.819602	7.2150558
